# Recurrent Cellulitis Presenting with Unilateral Leg Edema Mimicking Filarial Elephantiasis

**DOI:** 10.4269/ajtmh.25-0587

**Published:** 2026-03-10

**Authors:** Manasvin Onwan, Kantarat Wattanawinitchai, Sakarn Charoensakulchai

**Affiliations:** ^1^ Department of Preventive and Social Medicine, Faculty of Medicine, Srinakharinwirot University, Nakhon-Nayok, Thailand;; ^2^ Department of Internal Medicine, Faculty of Medicine, Srinakharinwirot University, Nakhon-Nayok, Thailand;; ^3^ Hospital for Tropical Diseases, Faculty of Tropical Medicine, Mahidol University, Bangkok, Thailand

## INTRODUCTION

**Figure 1.**
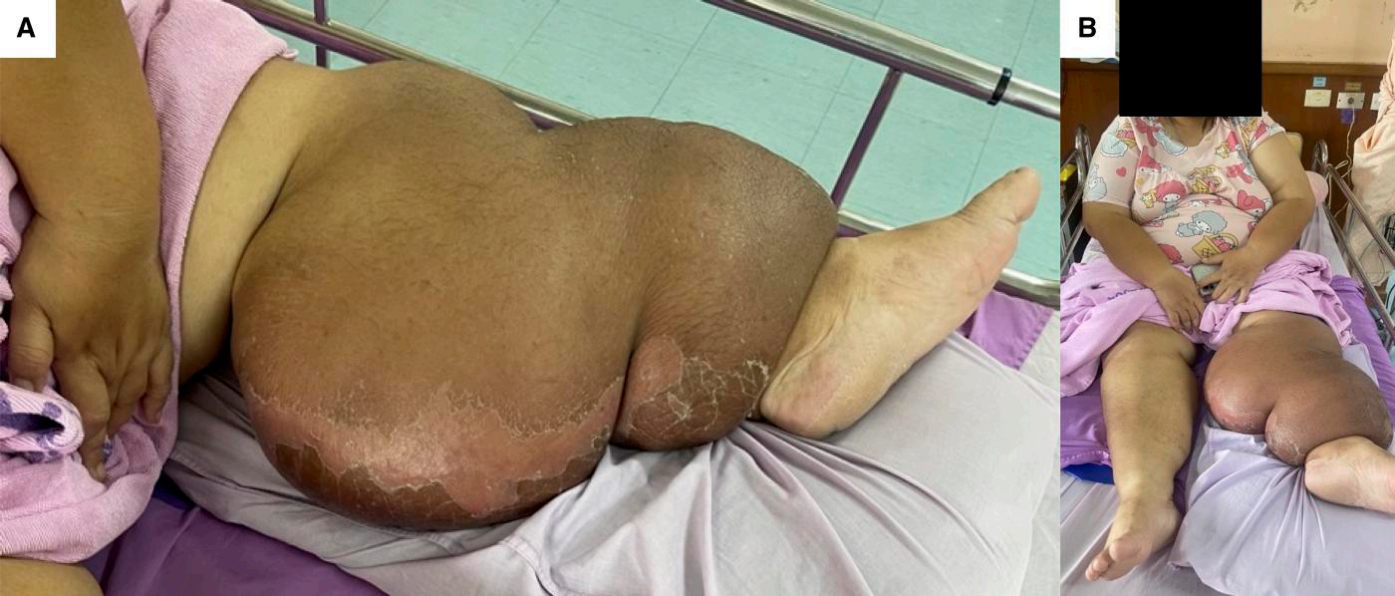
(**A**) Unilateral left leg edema. (**B**) Comparison between the left and right legs.

A 37-year-old Thai woman with morbid obesity presented with fever and enlargement of the left leg, extending from below the knee to the ankle, featuring hyperpigmentation and superficial desquamation (Figure 1). She had experienced more than five episodes of recurrent cellulitis within the past year, with the latest episode complicated by the development of drug-resistant infection. She had no history of surgery or trauma around that extremity. Initially, lymphatic filariasis was suspected. Microfilariae were not observed on three consecutive nighttime blood smears. Anti-filarial antibody (IgG4) testing results were negative. A Doppler ultrasound revealed no evidence of deep venous thrombosis and no “filarial dancing sign.” A skin biopsy revealed superficial and deep perivascular lymphoplasmacytic infiltration with fibrosis. A complete blood count revealed leukocytosis with neutrophil predominance, without eosinophilia. Chest radiography revealed no pulmonary lesions. A computed tomography scan of the lower extremity and lymphoscintigraphy were not obtained because of the patient’s financial constraints. It was concluded that the patient had lymphedema secondary to recurrent cellulitis with predisposing obesity.

Secondary lymphedema has multiple causes, with lymphatic filariasis being the most common in tropical, resource-limited settings.^1^^,^^2^ Despite the certified elimination of lymphatic filariasis in Thailand in 2017, reports of imported cases among migrants and indigenous cases are occasionally reported.^3^ Therefore, a local underreported transmission was possible, prompting the work-up on lymphatic filariasis. In this patient, recurrent cellulitis and obesity likely contributed to pathogenesis. Obesity increases lymphatic fluid production and impairs drainage by causing adipose tissue compression.^1^ Consequently, the preexisting minimal level of lymphedema can predispose patients to cellulitis because the stagnated and leaked lymphatic fluid can serve as a medium for bacterial proliferation and cause local immunity to be compromised.^1^^,^^4^^,^^5^ Conversely, cellulitis damages the surrounding lymphatic vessels, thus further impairing lymphatic drainage.^1^ When this vicious cycle is repeated without a cure, the progression of lymphedema can occur. Filarial elephantiasis usually causes chronic limb swelling, with thickened, hyperpigmented skin. Although this patient exhibited similar findings due to recurrent cellulitis, the condition’s clinical mimicry to lymphatic filariasis highlights the need to consider recurrent cellulitis as a cause of lymphedema, particularly in filariasis-endemic areas, to ensure accurate diagnosis and appropriate management.
